# Fire and summer temperatures interact to shape seed dormancy thresholds

**DOI:** 10.1093/aob/mcac047

**Published:** 2022-04-07

**Authors:** Maya Zomer, Bruno Moreira, Juli G Pausas

**Affiliations:** Centro de Investigaciones sobre Desertificación (CIDE-CSIC), Ctra. Náquera Km. 4.5, Moncada, E-46113 Valencia, Spain; Centro de Investigaciones sobre Desertificación (CIDE-CSIC), Ctra. Náquera Km. 4.5, Moncada, E-46113 Valencia, Spain; Centro de Investigaciones sobre Desertificación (CIDE-CSIC), Ctra. Náquera Km. 4.5, Moncada, E-46113 Valencia, Spain

**Keywords:** Fire, summer, Mediterranean, Cistaceae, *Cistus*, physical seed dormancy, germination

## Abstract

**Background and Aims:**

In Mediterranean ecosystems, the heat shock of wildfire disrupts physical seed dormancy in many plant species. This triggers germination in the post-fire environment where seedling establishment is optimal due to decreased competition and increased resource availability. However, to maintain the soil seed bank until a fire occurs, the minimum heat capable of breaking seed dormancy (i.e. the lower heat threshold) must be above the maximum temperatures typically observed in the soil during the summer. We therefore hypothesized that summer temperatures have shaped heat requirements for physical dormancy release. Specifically, we predicted that seeds from populations growing under warmer summers will have higher values of the lower heat threshold.

**Methods:**

To evaluate this prediction, we collected seeds from two *Cistus* species in 31 populations (20 *Cistus albidus* and 11 *Cistus salviifolius*) along a climate gradient of summer temperatures on the eastern coast of Spain. For each population, seeds were treated to 10 min heat shocks, from 30 to 120 °C in 5 °C increments (19 treatments), to simulate increasing heat doses from summer to fire-related temperatures. Seeds were then germinated in the lab.

**Key Results:**

For all populations, maximum germination was observed when applying temperatures associated with fire. Lower heat thresholds varied among populations, with a positive relationship between summer temperatures at seed population origin and the heat dose required to break dormancy.

**Conclusions:**

Our results suggest that fire drives maximum dormancy release for successful post-fire germination, while summer temperatures determine lower heat thresholds for ensuring inter-fire seed bank persistence. Significant among-population variation of thresholds also suggests that post-fire seeder species have some potential to modify their dormancy release requirements in response to changing climate.

## INTRODUCTION

Mediterranean ecosystems are dominated by flammable vegetation composed of plant species that have evolved to persist with hot, dry summers and recurrent fires (Keeley *et al*., 2011, 2012). Seed dormancy is a key adaptive trait especially prominent in ecosystems with periodic disturbances (Gioria *et al.*, 2020). Its function is to prevent germination until specific external stimuli signal that conditions are favourable for seedling establishment (Thompson and Ooi, 2010; Baskin and Baskin, 2014). There is extensive evidence that both heat shocks and smoke from wildfires break seed dormancy and stimulate germination in a wide range of species (Keeley and Fotheringham, 2000; Moreira *et al.*, 2010; Pausas and Lamont, 2022). Heat-released dormancy occurs in physically dormant seeds that form long-lived, fire-resistant soil seed banks, and have temperature requirements for breaking dormancy that typically match fire heat (Moreira and Pausas, 2012; Pausas and Lamont, 2022). These seeds are hard and impermeable, with special structures in the seed coat that are disrupted only when exposed to high temperatures, allowing water uptake (Baskin *et al.*, 2000; Gama-Arachchige *et al.*, 2013). Thus, the passage of fire breaks physical dormancy and enables germination during the following post-fire rains (post-fire seeding). Seed banks are also subject to seasonal soil heating by the sun; yet the role of hot summers in the evolution of heat-released physical dormancy is not fully understood (Ooi *et al.*, 2012; Santana *et al.*, 2013; Cochrane, 2017).

Releasing physical seed dormancy (hereafter, dormancy) by the heat of fire or by the heat of summer has different ecological consequences for species. Fire-released dormancy stimulates massive germination in a post-fire environment optimal for seedling growth (high water, light and nutrient availability, and reduced competition; Keeley and Fotheringham, 2000). Thus, fire-released dormancy undoubtedly provides an ecological advantage for obligate seeder species in fire-prone Mediterranean ecosystems (Keeley *et al.*, 2012). In contrast, summer-released dormancy would result in germination under sub-optimal conditions for seedling establishment (shade and high competition) and is generally a net loss to the seed bank (Keeley, 1987; Ooi *et al.*, 2012); thus, dormancy release thresholds are subject to strong selection pressure (Hudson *et al.*, 2015). Consequently, numerous studies have confirmed that a single, short duration, heat shock of fire temperatures is the most effective for maximum dormancy release (Auld and O’Connell, 1991; Keeley, 1991; Moreira and Pausas, 2012), while accumulated summer heat makes a minor contribution to dormancy release (reviewed in Pausas and Lamont, 2022). However, the persistence of seeds in the soil seed bank and therefore the resilience of post-fire seeder species relies on the minimum temperatures capable of breaking dormancy (hereafter, lower heat thresholds) being above soil temperatures experienced during summers. Thus, summer temperatures may have a role in shaping seed dormancy release thresholds. Indeed, previous studies have shown that heat wave temperatures at cooler (higher altitude) sites intensify dormancy release, suggesting that the climate under which the seeds develop determines their heat requirements (Ooi *et al.*, 2012). The current increase of summer temperatures and heat wave events due to global warming (Parmesan *et al.*, 2022) raises concerns that depletion of the seed bank during the coming summers could limit post-fire regeneration of many seeder species in Mediterranean ecosystems (Ooi *et al*., 2012, 2014; Cochrane, 2017). Disentangling the factors responsible for shaping lower heat thresholds (that allow seed bank persistence) from those responsible for driving maximum dormancy release (for optimal seedling establishment) may inform us on the vulnerability of the species to climatic changes.

We hypothesized that in fire-prone Mediterranean ecosystems, summer temperatures shape the lower heat threshold at which physical dormancy is released. Specifically, we predicted that seeds from populations with higher summer temperatures will have increased lower heat thresholds than those from populations inhabiting areas with lower summer temperatures. To test this prediction, we sampled populations of two *Cistus* species (Cistaceae) along a climatic gradient on the Mediterranean coast of Spain. *Cistus* species are widespread in the Mediterranean Basin, have seeds with physical dormancy, respond positively to experimental heat shock and massively regenerate post-fire (Moreira and Pausas, 2012; Tavsanoglu and Pausas, 2018).

## MATERIALS AND METHODS

### Species and climate gradient

Our species models are *Cistus albidus* and *Cistus salviifolius* (Cistaceae); both are shade intolerant, and grow in shrublands with a typical Mediterranean climate. Latitudinally, both species range from southern France to northern Africa. *Cistus albidus* is limited to the western Mediterranean basin, while the range of *C. salviifolius* covers the majority of the Mediterranean basin (Bolòs and Vigo, 1990). The two species are pollinated by insects and have short-distance dispersal (mainly autochory; Tavsanoglu and Pausas, 2018).

**Fig. 1.**
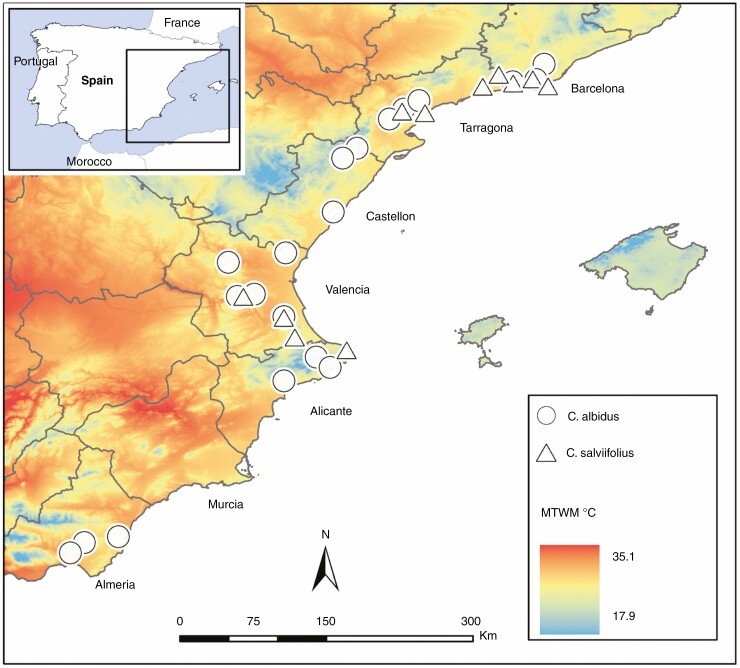
Location of the study area (boxed region) in the Iberian Peninsula, showing the gradient of the maximum temperature of warmest month (°C) (MTWM) and location of the study sites of *Cistus albidus* (*n* = 20) and *Cistus salviifolius* (*n* = 11); borders are provinces. Sources: CHELSA 1.2, 30 arc sec, 1979–2013 (Karger *et al*, 2017).

We selected sites with abundant *C. albidus* and/or *C. salviifolius* along the eastern coast of Spain, from Barcelona to Almeria, spanning a climate gradient (with contrasting temperatures, precipitation and aridity). Sites were chosen up to 1000 m elevation and with comparable habitat ‘openness’ (shrublands with minimal tree cover and avoiding shady microsites), although the nature of the climate gradient inevitably resulted in lower vegetation density and larger areas of bare ground in more arid sites. *Cistus albidus* were collected along the whole latitudinal gradient studied (approx. 600 km) while *C. salviifolius* only occurred in the northern half (approx. 300 km) of our gradient (Fig. 1).

For each site, we obtained bioclimatic variables derived from monthly mean and maximum temperature and precipitation values from the CHELSA 1.2 global dataset (Karger *et al*., 2017, 2018; 30 arc second spatial resolution for the years 1979–2013). Maximum temperature of warmest month and mean annual temperature were chosen to represent the role of temperature at seed origin in dormancy release, while mean annual precipitation and aridity were chosen to test other climate factors known to impact the development of seed traits (Cochrane *et al.*, 2014). The aridity index was calculated as the quotient between mean annual precipitation and mean annual potential evapotranspiration, with lower values indicative of more arid conditions (Zomer *et al.*, 2008).

An estimation of the recent fire history was determined from historical fire records provided by local governments (Junta de Andalucía, Generalitat Valenciana and Generalitat de Catalunya). Records were available from 1975 to 2016 for the province of Almeria; from 1976 to 2014 for the province of Valencia; from 1993 to 2014 for the provinces of Castellon and Alicante; and from 1987 to 2017 for the provinces of Tarragona and Barcelona. Our study region typically has intense crown fires concentrated within the summer months, with fire intervals in the order of decades. We only had available a maximum of 20–40 years of fire records, which is too short to correctly determine fire frequency for our sites. In addition, because our gradient has a short geographical range and is within the same vegetation type (shrublands), the variability in fire intensity is low and difficult to depict. However, burned area in the surroundings of each site is more variable and a good proxy for fire activity in the study area. Thus, we calculated the average area burned each year within a 10 km radius buffer circle surrounding each site. To account for sites with buffer circles that overlapped with the sea, we converted these values to the percentage of buffer area (hectares on land) burned each year. To ensure minimum fire presence, only sites with at least 0.1 % of the buffer burned annually were chosen; thus, our final sampling gradient includes 26 sites (Supplementary data Table S1). Bioclimatic variables and fire history values were extracted for sites and buffer circles using ArcGIS Pro 2.8 (https://www.esri.com/en-us/arcgis/products/arcgis-pro/overview).

### Seed collection and germination experiment

Seeds were collected from 20 *C. albidus* populations and 11 *C. salviifolius* populations along the 26 sites in the Mediterranean coast of Iberia (Fig. 1), that span a wide range of environmental conditions (Supplementary data Table S1). Mature capsules were collected from at least 20 individuals per species’ population, separated by a minimum distance of 5 m, between June and July 2018. Seeds were manually removed from fruits, and empty seeds were separated by density using a wind tunnel, followed by inspection under a magnifier to remove visibly damaged seeds. All seeds were stored in cellulose bags in a dark chamber at 20 °C for optimal storage conditions (Luna *et al.*, 2007; Saura-Mas *et al.*, 2020). There is evidence that seed mass varies along environmental gradients (Cochrane *et al.*, 2014), and that seed mass influences germination response to heat shock and heat resistance among species of post-fire seeders (Hanley *et al.*, 2003; Liyanage and Ooi, 2017), and within populations of *Cistus* species (Tavşanoğlu and Çatav, 2012). Thus, we weighed ten seeds from each population to include seed mass in our analysis.

The final germination experiment was carried out in February 2020 and consisted of the exposure of seeds to a range of 10 min dry heat shock treatments in a pre-heated oven, from 30 to 120 °C in 5 °C increments (19 treatments). This heat range was chosen to simulate increasing heat doses reflecting temperatures similar to those that occur in the superficial layers of the soil during the summer (up to 50 °C in open gaps) and during fire (over 80 °C). Seed banks of post-fire seeders in Mediterranean ecosystems are usually located in the upper 5 cm of soil (with most seeds in the top 2.5 cm) (Odion and Davis, 2000; Clemente *et al.*, 2007; Lamont *et al.*, 2022). Fire temperatures at this depth typically reach over 80 °C (Trabaud, 1979; Santana *et al.*, 2013), while registered soil temperatures at the peak of summer currently reach an average maximum of 40 °C and, briefly, absolute maximums of 50–60 °C (Brits, 1986; Baeza and Roy, 2008; Santana *et al.*, 2013). Experimental evidence has shown that seeds with physical dormancy respond to a range of temperatures, with germination increasing as temperatures increase, until heat-induced seed mortality begins to occur (Auld and O’Connell, 1991; Santana *et al.*, 2010; Hudson *et al.*, 2015). Heat shock treatments were applied separately to 20–25 seeds (depending on seed availability) for each population, per species, in three independent batch replicates (Morrison and Morris, 2000). Thus, >35 000 seeds were used for the experiment (31 populations × 19 treatments × 20–25 seeds × 3 replications). Directly after oven treatments, seeds were sown in Petri dishes of 5.5 cm lined with two layers of cotton and one sheet of filter paper, and moistened with 10 mL of distilled water. Petri dishes were sealed with parafilm and incubated in temperature- and humidity-controlled germination chambers (Sanyo MLR-350H) at 20 °C and 60 % humidity, in darkness. The total number of germinated seeds, determined by emergence of the radicle, was counted after 4 weeks. Before analysis, the initial number of seeds sown was corrected by discarding empty seeds (lacking embryo and storage tissue) detected during the experiment.

### Statistical analysis

Germination data were plotted against the 19 experimental heat temperatures to obtain a germination curve for each population. Germination typically increases with heat until a maximum is reached at temperatures optimal for dormancy release (typically 80–110 °C for *Cistus* species; Moreira and Pausas, 2012), then begins to fall due to an increase in seed mortality at very high temperatures. The lowest heat treatment of 30 °C, equivalent to the mean temperature during the summer, was used to standardize the germination curve. This temperature has been previously shown to not break seed dormancy for our species (Moreira and Pausas, 2012), therefore germination at this heat treatment represents the fraction of non-dormant seeds. For each population, we removed this non-dormant fraction from the final analysis. Maximum germination and the corresponding heat treatment were identified for each population.

**Fig. 2.**
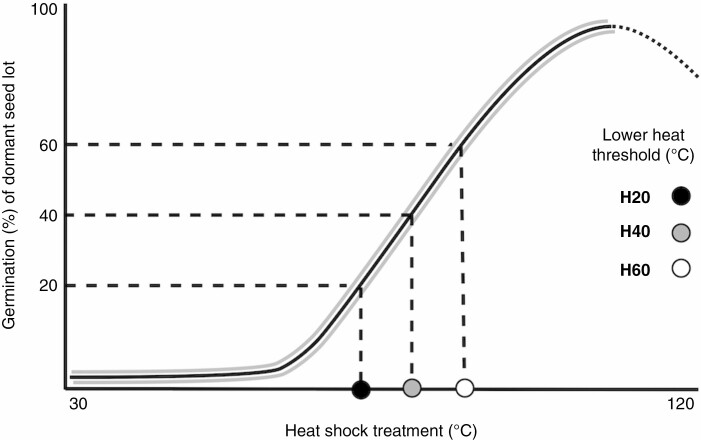
Schematic diagram showing the method for estimating lower dormancy release heat thresholds. A non-linear dose–response curve (solid line with 95 % confidence interval bands) was fitted to germination data of each population to predict, for a fixed percentage of dormancy release (i.e. 20, 40 and 60 %), the minimum heat dose needed (H20, H40 and H60, respectively).

We assessed the relationship of population-specific germination response and experimental heat treatments using dose–response curves (Fig. 2) as implemented in the ‘drc’ package (Ritz *et al.*, 2015) in the R statistical environment (R Core Team, 2020). To do so, we used the first part of the germination curve, i.e. up to the maximum (for each population), and fitted either a four-parameter non-linear Weibull or a four-parameter log-logistic function, chosen according to the Akaike information criterion (AIC), log likelihood estimations and lack-of-fit test (Supplementary data Table S2). This non-linear curve approach was chosen as it does not compress natural variance of the data, and fits only the parameters necessary for the model, thereby reducing the risk of overfitting (Ritz *et al.*, 2013; Tangney *et al.*, 2019; Rajapakshe *et al.*, 2020). Four parameters were necessary as the lower limits were not fixed at 0 after standardizing the curve for non-dormant seeds (there were a range of low heat treatments that likely caused mortality in non-dormant seeds, but didn’t stimulate germination in dormant ones). From these models, the effective dose (ED) function was used to predict the heat dose required for the response of seeds to reach the 20th, 30th, 40th, 50th and 60th percentiles of germination, with 95 % confidence intervals (Fig. 2; Supplementary data Table S3). We considered these percentages as absolute values, rather than relative as is the default in the ED function, to facilitate the comparison between populations. These effective heat doses describe the ‘lower heat thresholds’ at which 20–60 % dormancy release can occur for each population. In this analysis, percentage germination is equivalent to percentage dormancy release because all seeds had optimal conditions for germination and non-dormant seeds were previously removed from the analysis. Dormancy release of ≥20 % of the seed lot was considered a significant response to heat, as it is similar to seasonal dormancy release of hard-seeded species in non-fire-prone habitats (Ooi *et al.*, 2014). Dormancy release of 60 % of the seed lot was the maximum percentile with sufficient ED estimates for analysis, as several populations reached their maximum germination between 60 and 70 % of the seed lot.

To test to what extent heat thresholds of each population were related to their local climate, a linear regression model was fitted to lower heat threshold estimates. Species, dormancy release level, latitude, bioclimatic, fire and seed mass variables were added to the model as fixed effects. We used a stepwise approach to select the most parsimonious model; each step was assessed with analysis of variance (ANOVA) test and AIC. All analyses were carried out in the R statistical environment (R Core Team 2020).

## RESULTS

**Fig. 3.**
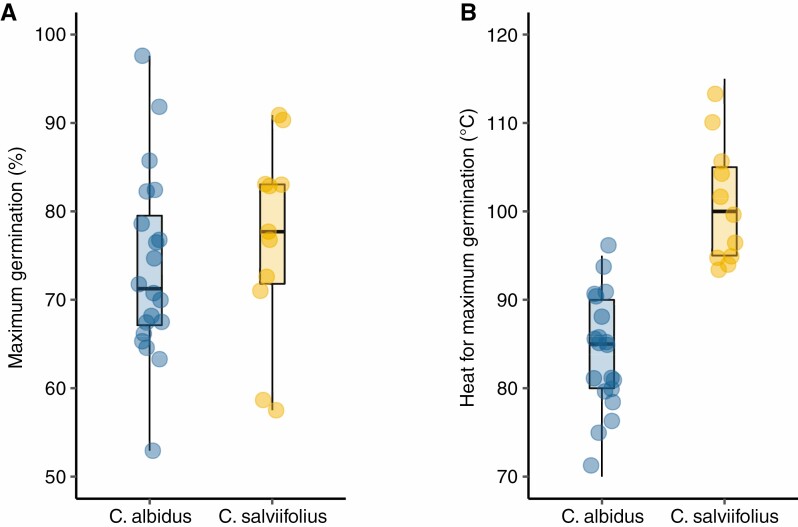
For the two species studied, *Cistus albidus* and *Cistus salviifolius*: (A) Maximum germination reached during the experiment; and (B) experimental heat treatment (°C) required to stimulate maximum germination. For each species, variability refers to across populations (circles).

Maximum germination varied greatly among populations, ranging from approx. 55 % to approx. 95 % for both species (Fig. 3A). Experimental heat treatments required to stimulate maximum germination for each population were always higher than 70 °C (mean of 83.7 °C and 100.9 °C for *C. albidus* and *C. salviifolius*, respectively; Fig. 3B), i.e. temperatures associated with fire.

**Fig. 4.**
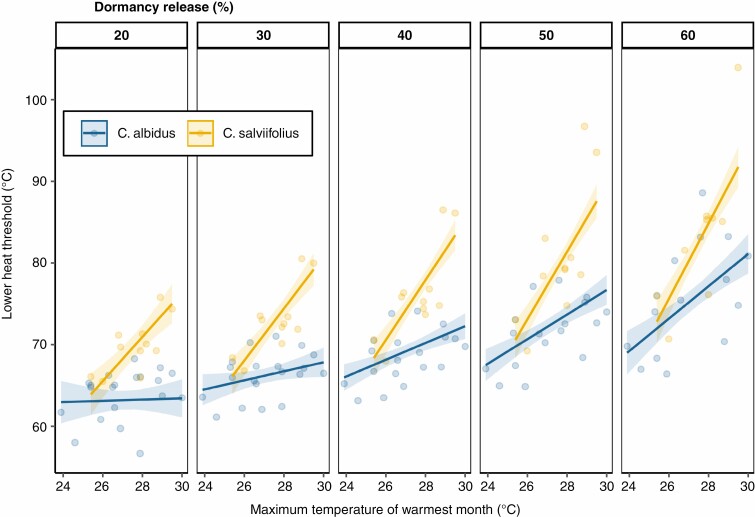
Lower heat thresholds (°C, *y*-axis) for 20–60 % dormancy release values (separated by panels), in relation to maximum summer temperatures of the seed population origin (°C, *x*-axis) for the two species studied, *Cistus albidus* and *Cistus salviifolius*. Lines are predicted values, bands are confidence intervals of the linear regression model (Table 1) and circles are the raw data (20–60 % dormancy release: *n* = 31; *n* = 31; *n* = 30; *n* = 28; n = 24). For statistical significance, see Table 1.

Dormancy release level, species and mean maximum temperature of warmest month (summer temperature) were the variables that best explained lower heat thresholds (Table 1). Once these variables were in the model, none of the other variables tested (latitude, mean annual temperature, mean annual precipitation, aridity index, fire history and seed mass) were entered in the model. There was a significant positive association in which the higher the summer temperature at seed population origin, the higher the lower heat threshold (Fig. 4). The interaction with summer temperature was significant for both species and dormancy release level, suggesting that the two species behave differently and that the influence of summer temperature differs in strength at the different levels of dormancy release (Fig. 4). Overall, *C. salviifolius* had higher heat thresholds, greater variability of lower heat thresholds (across populations) and stronger response to summer temperatures than *C. albidus*. The range of threshold variability in response to summer temperature increased for both species as the dormancy release level increased from 20 to 60 % (Fig. 4). Predicted heat thresholds were in all cases above 60 °C, and therefore above current summer temperatures.

**Table 1.** Summary of the linear regression model for population lower heat thresholds (°C) in relation to maximum temperature of warmest month, percentage dormancy release and species

Model	d.f.	AIC	*P*	Estimate
Null		990.7431		80.939
+ Dormancy release (%)	1	931.8578	<0.0001	–0.990
+ Species	1	872.3845	<0.0001	–66.132
+ Maximum temperature of warmest month (MTWM)	1	831.2089	<0.0001	–0.088
+ MTWM × Dormancy release (%)	1	824.1229	0.003	0.005
+ MTWM × Species	1	798.5907	<0.0001	0.263

The variables included in the final model were selected by the stepwise procedure. Models were compared with ANOVA test and the Akaike information criterion (AIC). Model statistics for each are presented: *n* = 144. Note that each row represents an individual model. The last column provides coefficient estimates for the intercept (row of Null model) and each fixed effect of the final model. This final model is displayed in Fig. 4.

## DISCUSSION

Seed dormancy is a mechanism to ensure that germination occurs in the optimal conditions for seedling establishment (Baskin and Baskin, 2014). In Mediterranean ecosystems, where shrub cover is high, the optimal conditions for the germination of many species are after fire (low competition and high resources) (Pausas and Lamont, 2022). In agreement with that, we found that maximum dormancy release for all populations of both study species occurred at temperatures related to fire (Fig. 3B).

However, for maximizing fire-released dormancy, seeds need to maintain dormancy during the inter-fire period, as germinating in such conditions would be a loss of opportunity (low light, high competition that prevents establishment) (Ooi *et al.*, 2012). Our results showed significant variation of dormancy release heat thresholds among populations, with lower heat thresholds positively associated with historical summer temperatures of seed origin (Table 1; Fig. 4), i.e. seeds from populations with higher summer temperatures required more heat for the same percentage of dormancy release. Additionally, predicted heat thresholds needed to reach dormancy release levels of 20–60 % were above 60 °C and therefore above current summer temperatures registered on bare soil. The highest recorded summer soil maximums in the Mediterranean Basin are 50–60 °C in open gaps (Baeza and Roy, 2008; Santana *et al.*, 2013). It is worth noting that those maximum temperatures are infrequent and would normally occur only in soil exposed either by fire or by anthropogenic gaps, not in unburnt natural shrubland (Moreira and Pausas, 2012; Lamont *et al.*, 2022). Our results therefore support the idea that climate is the key determinant in shaping lower dormancy release thresholds, for inter-fire seed bank persistence. The magnitude of threshold variation and strength of the regression coefficient with summer temperatures increased significantly with higher levels of dormancy release (Table 1; Fig. 4), i.e. the selective pressure of summer temperatures is stronger for thresholds capable of stimulating 60 % dormancy release of the seed bank than it is at thresholds for 20 %, because the risk of seed bank depletion from untimely germinations is more severe. Species may afford allocation of a small proportion of seeds for bet-hedging recruitment in inter-fire periods, but must ensure that most seeds are saved in the seed bank for post-fire recruitment (i.e. to ensure environmental matching for recruitment; Pausas and Lamont, 2022).

Intraspecific variation of traits is an important factor in the ability of a species to adapt to short-term environmental change (Ooi *et al*., 2012, 2014; Chamorro *et al.*, 2018). Predictions for the Mediterranean coast of Spain are for an increase in maximum summer temperatures (Zittis *et al.*, 2019) and in the frequency and intensity of heatwaves (Lorenzo and Alvarez, 2020; Parmesan *et al.*, 2022). These changes will probably be uneven across a species’ distribution (Cochrane *et al.*, 2014). Among-population variability of seed traits along geographic and climate gradients is an indicator of either genetic variation or plasticity driven by the parental environment (Roches *et al.*, 2018), and may provide species with the ability to adjust their phenotype or to shift their distribution range to remain in familiar environmental conditions (Alberto *et al.*, 2013; Parmesan and Hanley, 2015; Henn *et al.*, 2018). For post-fire seeder species whose persistence depends on the maintenance of the soil seed bank until the arrival of fire, variation in dormancy release thresholds is an indicator of their resilience to rising summer temperatures. We showed large differences in threshold temperatures between populations at the extremes of the gradient; *C. salviifolius* had a range of 11.1 °C and 19 °C of predicted thresholds for 20 % and 60 % dormancy release, respectively, while the range of these predicted thresholds was lower for *C. albidus* (0.5 °C and 12.1 °C for 20 % and 60 % dormancy release, respectively), despite having a longer sampling gradient (Fig. 4). Characteristics of seed morphology are likely to be the underlying factors in regulating heat thresholds and driving the observed differences among populations and species (Liyanage and Ooi, 2017). Seed mass did not explain variability of heat thresholds in our study, but seed coat thickness has been identified as a possible factor and warrants further study (Lamont *et al.*, 2021, Preprint).

The presence of significant among-population variability in dormancy release thresholds suggests that our study species have the potential to respond to environmental changes. However, *C. albidus* had relatively lower thresholds and less variation, with predicted thresholds as low as 63 °C for 20 % dormancy release. This indicates that although the overall trend is the same, patterns of threshold variation are idiosyncratic between species. While *C. albidus* does have some potential for readjusting thresholds, this species may be more vulnerable to seed bank depletion during future summer heat events, with potentially negative implications for post-fire regeneration. To what extent this vulnerability is modified by variability of seed burial depth in the field, which in turn depends on soil type, soil texture, seed size or time since dispersal (Tangney *et al.*, 2020), requires further research. Our results also suggest that selecting seeds from seeder populations with high dormancy release heat thresholds for restoration projects would be likely to increase the resilience capacity of the restored system to warmer climates (Parmesan and Hanley, 2015).

In conclusion, fire is the driver of maximum dormancy release for optimal seedling recruitment, but to ensure inter-fire bank persistence, the lower thresholds of heat capable of breaking dormancy are driven by summer temperatures. There is considerable among-population variability of thresholds across the summer temperature gradient, highlighting the potential for post-fire seeder species to modify dormancy release requirements in response to global climate change. Furthermore, lower dormancy release thresholds can be used as an indicator of the vulnerability of the species to global warming.

## SUPPLEMENTARY DATA

Supplementary data are available online at https://academic.oup.com/aob and consist of the following. Table S1: seed population number, site number, province, and site characteristics for *C. salviifolius* and *C. albidus*. Table S2: model description for germination dose–response curves for *C. salviifolius* and *C. albidus*. Table S3: estimated effective doses for the 20th–60th percentiles of dormancy release for *C. salviifolius* and *C. albidus*.

mcac047_suppl_Supplementary_Material
