# Supplementary material for: Fire and summer temperatures interact to shape seed dormancy thresholds
Source: Ann Bot. 2022 Apr 7;129(7):809–16. doi: 10.1093/aob/mcac047 (PMC9292603; doi:10.1093/aob/mcac047)
Supplement: mcac047_suppl_Supplementary_Material [file mcac047_suppl_supplementary_material.docx]

**Supplementary Materials**

Zomer M, Moriera B, Pausas JG. Fire and summer temperatures interact to shape seed dormancy thresholds. *Annals of Botany*

**Table S1.** Seed population number, site number, and province (Eastern Spain) sorted by latitude for *C. salviifolius* (CSA) and *C. albidus* (CAL). Site characteristics include elevation, annual mean temperature, maximum temperature of warmest month, and annual precipitation (obtained from CHELSA v1.2, 1979 – 2016), aridity index (lower values indicate more arid conditions), mean percentage of buffer circle burned annually (see methods for calculation), and seed mass in grams.

| **Species** | **Population number (*Sit*e)** | **Province** | **Latitude** | **Longitude** | **Elevation (m)** | **Annual mean temperature [°C]** | **Maximum temperature of warmest month [°C]** | **Annual precipitation [mm/year]** | **Aridity index** | **Mean % of buffer circle burned annually** | **Seed mass of 10 seeds * 100 [g]** |
| --- | --- | --- | --- | --- | --- | --- | --- | --- | --- | --- | --- |
| **CSA** | 1 (1) | Alicante | 38.809761 | 0.162458 | 42 | 17.2 | 26 | 625 | 1.05 | 0.797 | 1.007 |
| **CSA** | 2 (*2*) | Valencia | 38.92775 | -0.314389 | 392 | 15.9 | 26.9 | 637 | 0.75 | 1.663 | 0.957 |
| **CSA** | 3 (*3*) | Valencia | 39.107848 | -0.414571 | 201 | 17.1 | 28.9 | 735 | 0.78 | 1.273 | 0.88 |
| **CSA** | 4 (*4*) | Valencia | 39.300432 | -0.788097 | 778 | 13.6 | 27.9 | 637 | 0.63 | 5.910 | 1.083 |
| **CSA** | 5 (*5*) | Tarragona | 40.987378 | 0.882237 | 118 | 16 | 28.7 | 524 | 0.55 | 0.858 | 1.055 |
| **CSA** | 6 (*6*) | Tarragona | 41.002028 | 0.671214 | 148 | 16.2 | 29.5 | 574 | 0.58 | 0.852 | 1.093 |
| **CSA** | 7 (*7*) | Tarragona | 41.229922 | 1.412922 | 245 | 15.4 | 28.2 | 645 | 0.69 | 0.467 | 1.161 |
| **CSA** | 8 (*8*) | Barcelona | 41.259764 | 1.692788 | 182 | 15.8 | 28 | 631 | 0.71 | 0.332 | 1.013 |
| **CSA** | 9 (*9)* | Barcelona | 41.262878 | 1.687627 | 261 | 15.6 | 27.9 | 660 | 0.74 | 0.319 | 1.098 |
| **CSA** | 10 (*10*) | Barcelona | 41.283547 | 1.903944 | 393 | 13.8 | 25.4 | 799 | 1.01 | 0.838 | 1.079 |
| **CSA** | 11 (*11*) | Barcelona | 41.297602 | 1.872988 | 385 | 15 | 26.8 | 691 | 0.83 | 0.897 | 1.106 |
| **CAL** | 1 (*12*) | Almeria | 37.018201 | -2.266905 | 1036 | 13.2 | 25.3 | 365 | 0.41 | 0.196 | 1.197 |
| **CAL** | 2 (*13*) | Almeria | 37.032905 | -2.24709 | 699 | 13.9 | 25.9 | 345 | 0.38 | 0.228 | 0.947 |
| **CAL** | 3 (*14*) | Almeria | 37.08721 | -1.933791 | 399 | 14.2 | 25.4 | 344 | 0.40 | 1.153 | 0.849 |
| **CAL** | 4 (*15*) | Alicante | 38.516764 | -0.41339 | 551 | 15.2 | 26.3 | 451 | 0.52 | 0.125 | 1.027 |
| **CAL** | 5 (*16*) | Alicante | 38.675257 | -0.036586 | 620 | 14.6 | 23.9 | 610 | 0.95 | 0.481 | 0.876 |
| **CAL** | 6 (*17)* | Alicante | 38.735584 | -0.119168 | 563 | 14.8 | 24.6 | 661 | 0.94 | 0.421 | 1.114 |
| **CAL** | 7 (*3*) | Valencia | 39.107848 | -0.414571 | 201 | 17.1 | 28.9 | 735 | 0.78 | 1.273 | 1.009 |
| **CAL** | 8 (*4*) | Valencia | 39.300432 | -0.788097 | 778 | 13.6 | 27.9 | 637 | 0.63 | 5.910 | 0.876 |
| **CAL** | 9 (*18*) | Valencia | 39.313833 | -0.685972 | 366 | 16.2 | 30 | 573 | 0.54 | 3.902 | 1.026 |
| **CAL** | 10 (*19*) | Valencia | 39.606128 | -0.925097 | 438 | 13.9 | 29 | 490 | 0.47 | 3.648 | 1.096 |
| **CAL** | 11 (*20*) | Valencia | 39.694013 | -0.397524 | 575 | 14.5 | 26.9 | 609 | 0.67 | 1.084 | 1.03 |
| **CAL** | 12 (*21*) | Castellon | 40.067325 | 0.040802 | 116 | 16.4 | 27.7 | 531 | 0.61 | 0.385 | 0.889 |
| **CAL** | 13 (*22*) | Castellon | 40.564859 | 0.124883 | 613 | 13.4 | 26.5 | 495 | 0.54 | 0.598 | 1.18 |
| **CAL** | 14 (*23*) | Castellon | 40.601833 | 0.180121 | 456 | 13.5 | 26.6 | 491 | 0.53 | 0.615 | 1.181 |
| **CAL** | 15 (*6*) | Tarragona | 41.002028 | 0.671214 | 148 | 16.2 | 29.5 | 574 | 0.58 | 0.852 | 1.173 |
| **CAL** | 16 (*24*) | Tarragona | 41.012918 | 0.686946 | 269 | 15.5 | 28.8 | 606 | 0.62 | 0.637 | 1.292 |
| **CAL** | 17 (*25*) | Tarragona | 41.025345 | 0.709221 | 385 | 14.4 | 27.6 | 667 | 0.71 | 0.488 | 1.145 |
| **CAL** | 18 (*9*) | Barcelona | 41.262878 | 1.687627 | 261 | 15.6 | 27.9 | 660 | 0.74 | 0.319 | 1.112 |
| **CAL** | 19 (*10*) | Barcelona | 41.283547 | 1.903944 | 393 | 13.8 | 25.4 | 799 | 1.01 | 0.838 | 1.254 |
| **CAL** | 20 (*26*) | Barcelona | 41.369979 | 1.950343 | 282 | 14.7 | 26.6 | 653 | 0.79 | 0.247 | 1.071 |

**Table S2**. Model description for germination dose response curves (germination against experimental heat temperatures) for C. salviifolius (CSA) and C. albidus (CAL). The most parsimonious model was identified for each population using the mselect function within ‘drc’ package, to select the model with the lowest AIC and highest Log-likelihood. Model selection was either a four parameter Log-logistic model (LL.4, LL2.4) or a four parameter Weibull model (W1.4, W2.4). Final model selections are in bold.

| **Species** | **Population** | **Model** | **LogLik** | **AIC** | **Lack of fit** | **Res var** |
| --- | --- | --- | --- | --- | --- | --- |
| **CSA** | 1 | **W1.4** | **44.780** | **-79.560** | **0.396** | **0.008** |
|  |  | LL.4 | 44.469 | -78.938 | 0.359 | 0.008 |
|  |  | LL2.4 | 44.469 | -78.938 | 0.359 | 0.008 |
|  |  | W2.4 | 43.631 | -77.262 | 0.269 | 0.008 |
| **CSA** | 2 | **W2.4** | **34.110** | **-58.221** | **0.701** | **0.013** |
|  |  | LL.4 | 33.677 | -57.355 | 0.637 | 0.013 |
|  |  | LL2.4 | 33.677 | -57.355 | 0.637 | 0.013 |
|  |  | W1.4 | 33.140 | -56.281 | 0.559 | 0.013 |
| **CSA** | 3 | **W1.4** | **41.692** | **-73.384** | **1.000** | **0.015** |
|  |  | LL.4 | 41.103 | -72.206 | 0.999 | 0.015 |
|  |  | LL2.4 | 41.065 | -72.131 | 0.999 | 0.015 |
|  |  | W2.4 | 40.227 | -70.454 | 0.994 | 0.015 |
| **CSA** | 4 | **W1.4** | **32.970** | **-55.940** | **0.519** | **0.013** |
|  |  | LL.4 | 32.204 | -54.407 | 0.415 | 0.014 |
|  |  | LL2.4 | 32.204 | -54.407 | 0.415 | 0.014 |
|  |  | W2.4 | 31.423 | -52.846 | 0.322 | 0.014 |
| **CSA** | 5 | **W1.4** | **28.976** | **-47.951** | **0.818** | **0.019** |
|  |  | LL2.4 | 28.571 | -47.142 | 0.770 | 0.019 |
|  |  | LL.4 | 28.571 | -47.142 | 0.770 | 0.019 |
|  |  | W2.4 | 27.989 | -45.978 | 0.697 | 0.020 |
| **CSA** | 6 | **W1.4** | **33.268** | **-56.535** | **0.951** | **0.017** |
|  |  | LL.4 | 32.877 | -55.755 | 0.928 | 0.018 |
|  |  | LL2.4 | 32.877 | -55.755 | 0.928 | 0.018 |
|  |  | W2.4 | 32.404 | -54.808 | 0.892 | 0.018 |
| **CSA** | 7 | **W1.4** | **50.903** | **-91.805** | **0.827** | **0.008** |
|  |  | LL.4 | 49.200 | -88.400 | 0.612 | 0.008 |
|  |  | LL2.4 | 49.200 | -88.400 | 0.612 | 0.008 |
|  |  | W2.4 | 47.702 | -85.404 | 0.420 | 0.009 |
| **CSA** | 8 | **W2.4** | **23.250** | **-36.501** | **0.033** | **0.023** |
|  |  | LL.4 | 23.036 | -36.072 | 0.030 | 0.023 |
|  |  | LL2.4 | 23.036 | -36.072 | 0.030 | 0.023 |
|  |  | W1.4 | 22.996 | -35.992 | 0.030 | 0.023 |
| **CSA** | 9 | **W1.4** | **42.322** | **-74.644** | **0.444** | **0.010** |
|  |  | LL.4 | 41.577 | -73.155 | 0.355 | 0.010 |
|  |  | LL2.4 | 41.577 | -73.155 | 0.355 | 0.010 |
|  |  | W2.4 | 40.827 | -71.654 | 0.277 | 0.010 |
| **CSA** | 10 | **W1.4** | **29.654** | **-49.309** | **0.543** | **0.017** |
|  |  | LL2.4 | 28.745 | -47.490 | 0.424 | 0.018 |
|  |  | LL.4 | 28.745 | -47.490 | 0.424 | 0.018 |
|  |  | W2.4 | 27.257 | -44.513 | 0.262 | 0.019 |
| **CSA** | 11 | **W1.4** | **45.764** | **-81.528** | **0.365** | **0.008** |
|  |  | LL.4 | 45.092 | -80.185 | 0.293 | 0.009 |
|  |  | LL2.4 | 45.092 | -80.185 | 0.293 | 0.009 |
|  |  | W2.4 | 43.719 | -77.438 | 0.179 | 0.009 |
| **CAL** | 1 | **W1.4** | **12.107** | **-14.214** | **0.075** | **0.032** |
|  |  | LL.4 | 11.747 | -13.494 | 0.063 | 0.033 |
|  |  | LL2.4 | 11.747 | -13.494 | 0.063 | 0.033 |
|  |  | W2.4 | 11.355 | -12.709 | 0.051 | 0.033 |
| **CAL** | 2 | **W1.4** | **20.740** | **-31.479** | **0.127** | **0.021** |
|  |  | LL.4 | 19.898 | -29.796 | 0.086 | 0.022 |
|  |  | LL2.4 | 19.898 | -29.796 | 0.086 | 0.022 |
|  |  | W2.4 | 18.473 | -26.947 | 0.043 | 0.024 |
| **CAL** | 3 | **W2.4** | **32.378** | **-54.756** | **0.560** | **0.014** |
|  |  | LL.4 | 32.044 | -54.089 | 0.512 | 0.014 |
|  |  | LL2.4 | 32.044 | -54.089 | 0.512 | 0.014 |
|  |  | W1.4 | 31.468 | -52.936 | 0.433 | 0.014 |
| **CAL** | 4 | **W1.4** | **17.567** | **-25.133** | **0.234** | **0.025** |
|  |  | LL.4 | 17.308 | -24.615 | 0.211 | 0.025 |
|  |  | LL2.4 | 17.307 | -24.614 | 0.210 | 0.025 |
|  |  | W2.4 | 17.291 | -24.582 | 0.209 | 0.025 |
| **CAL** | 5 | **LL.4** | **30.481** | **-50.962** | **0.859** | **0.015** |
|  |  | LL2.4 | 30.481 | -50.962 | 0.859 | 0.015 |
|  |  | W1.4 | 30.384 | -50.768 | 0.848 | 0.015 |
|  |  | W2.4 | 30.337 | -50.673 | 0.843 | 0.015 |
| **CAL** | 6 | **LL.4** | **29.904** | **-49.809** | **0.415** | **0.014** |
|  |  | W1.4 | 24.548 | -39.097 | 0.045 | 0.019 |
|  |  | W2.4 | 23.549 | -37.099 | 0.028 | 0.020 |
|  |  | LL2.4 | 22.814 | -35.628 | 0.019 | 0.020 |
| **CAL** | 7 | **W1.4** | **30.155** | **-50.310** | **0.790** | **0.012** |
|  |  | LL2.4 | 29.939 | -49.879 | 0.756 | 0.012 |
|  |  | LL.4 | 29.939 | -49.879 | 0.756 | 0.012 |
|  |  | W2.4 | 29.606 | -49.212 | 0.702 | 0.013 |
| **CAL** | 8 | **W2.4** | **16.036** | **-22.072** | **0.543** | **0.029** |
|  |  | W1.4 | 15.969 | -21.938 | 0.533 | 0.029 |
|  |  | LL.4 | 15.956 | -21.912 | 0.531 | 0.029 |
|  |  | LL2.4 | 15.956 | -21.912 | 0.531 | 0.029 |
| **CAL** | 9 | **W1.4** | **20.760** | **-31.520** | **0.397** | **0.021** |
|  |  | LL.4 | 20.325 | -30.650 | 0.340 | 0.021 |
|  |  | LL2.4 | 20.325 | -30.650 | 0.340 | 0.021 |
|  |  | W2.4 | 20.146 | -30.292 | 0.318 | 0.022 |
| **CAL** | 10 | **W1.4** | **16.266** | **-22.531** | **0.448** | **0.028** |
|  |  | LL.4 | 15.863 | -21.725 | 0.394 | 0.029 |
|  |  | LL2.4 | 15.863 | -21.725 | 0.394 | 0.029 |
|  |  | W2.4 | 15.550 | -21.100 | 0.354 | 0.029 |
| **CAL** | 11 | **W1.4** | **19.791** | **-29.581** | **0.862** | **0.025** |
|  |  | LL.4 | 19.645 | -29.289 | 0.845 | 0.025 |
|  |  | LL2.4 | 19.645 | -29.289 | 0.845 | 0.025 |
|  |  | W2.4 | 19.495 | -28.991 | 0.827 | 0.026 |
| **CAL** | 12 | **W1.4** | **17.451** | **-24.902** | **0.781** | **0.028** |
|  |  | LL.4 | 17.412 | -24.825 | 0.776 | 0.028 |
|  |  | LL2.4 | 17.412 | -24.825 | 0.776 | 0.028 |
|  |  | W2.4 | 17.324 | -24.649 | 0.764 | 0.028 |
| **CAL** | 13 | **LL.4** | **3.110** | **3.781** | **0.358** | **0.056** |
|  |  | W1.4 | 3.092 | 3.816 | 0.356 | 0.056 |
|  |  | W2.4 | 3.079 | 3.841 | 0.354 | 0.056 |
|  |  | LL2.4 | 3.059 | 3.882 | 0.352 | 0.056 |
| **CAL** | 14 | **LL.4** | **28.188** | **-46.376** | **0.305** | **0.015** |
|  |  | LL2.4 | 28.188 | -46.376 | 0.305 | 0.015 |
|  |  | W2.4 | 28.177 | -46.355 | 0.304 | 0.015 |
|  |  | W1.4 | 28.172 | -46.344 | 0.303 | 0.015 |
| **CAL** | 15 | **W2.4** | **29.726** | **-49.452** | **0.228** | **0.019** |
|  |  | LL.4 | 29.332 | -48.663 | 0.198 | 0.019 |
|  |  | LL2.4 | 29.332 | -48.663 | 0.198 | 0.019 |
|  |  | W1.4 | 29.088 | -48.175 | 0.181 | 0.019 |
| **CAL** | 16 | **W1.4** | **17.614** | **-25.228** | **0.618** | **0.023** |
|  |  | LL.4 | 17.409 | -24.818 | 0.581 | 0.023 |
|  |  | LL2.4 | 17.407 | -24.814 | 0.581 | 0.023 |
|  |  | W2.4 | 17.027 | -24.053 | 0.515 | 0.024 |
| **CAL** | 17 | **W1.4** | **18.405** | **-26.810** | **0.089** | **0.025** |
|  |  | LL.4 | 17.590 | -25.180 | 0.061 | 0.026 |
|  |  | LL2.4 | 17.590 | -25.180 | 0.061 | 0.026 |
|  |  | W2.4 | 16.809 | -23.618 | 0.042 | 0.028 |
| **CAL** | 18 | **LL.4** | **3.188** | **3.624** | **0.870** | **0.055** |
|  |  | W2.4 | 3.179 | 3.641 | 0.869 | 0.055 |
|  |  | LL2.4 | 3.164 | 3.673 | 0.867 | 0.055 |
|  |  | W1.4 | 3.162 | 3.676 | 0.867 | 0.055 |
| **CAL** | 19 | **W2.4** | **25.843** | **-41.686** | **0.556** | **0.016** |
|  |  | LL.4 | 25.775 | -41.551 | 0.545 | 0.016 |
|  |  | W1.4 | 25.751 | -41.502 | 0.541 | 0.016 |
|  |  | LL2.4 | 25.670 | -41.339 | 0.528 | 0.016 |
| **CAL** | 20 | **W1.4** | **27.273** | **-44.547** | **0.090** | **0.016** |
|  |  | W2.4 | 27.240 | -44.479 | 0.088 | 0.016 |
|  |  | LL.4 | 27.072 | -44.145 | 0.082 | 0.016 |
|  |  | LL2.4 | 27.072 | -44.144 | 0.082 | 0.016 |

**Table S3.** Estimated effective doses (experimental heat) for the 20^th^ – 60^th^ percentiles of dormancy-release for *C. salviifolius* (CSA) and *C. albidus* (CAL), obtained from ‘ED’ function in drc package; considering percentages as absolute values, rather than relative as is the default in the *ED* function. (type = “absolute”); with 95% confidence intervals calculated using the delta method (interval = “delta”).

ie. ED (model, c(0.2, 0.3, 0.4, 0.5, 0.6), type = "absolute", interval = "delta")

| **Species** | **Population** | **Dormancy release %** | **Effective heat dose estimate (°C)** | **Standard Error** | **Lower** | **Upper** |
| --- | --- | --- | --- | --- | --- | --- |
| CSA | 1 | 20 | 65.490 | 0.794 | 63.882 | 67.098 |
|  |  | 30 | 66.792 | 0.654 | 65.468 | 68.116 |
|  |  | 40 | 67.976 | 0.613 | 66.735 | 69.217 |
|  |  | 50 | 69.224 | 0.683 | 67.841 | 70.606 |
|  |  | 60 | 70.699 | 0.881 | 68.915 | 72.482 |
| CSA | 2 | 20 | 69.691 | 2.039 | 65.563 | 73.820 |
|  |  | 30 | 73.017 | 1.683 | 69.610 | 76.424 |
|  |  | 40 | 76.351 | 2.118 | 72.063 | 80.638 |
|  |  | 50 | 83.019 | 4.288 | 74.339 | 91.699 |
|  |  | 60 |  |  |  |  |
| CSA | 3 | 20 | 75.791 | 2.185 | 71.409 | 80.173 |
|  |  | 30 | 80.512 | 3.119 | 74.257 | 86.767 |
|  |  | 40 | 86.490 | 5.094 | 76.274 | 96.707 |
|  |  | 50 | 96.715 | 9.217 | 78.228 | 115.201 |
|  |  | 60 |  |  |  |  |
| CSA | 4 | 20 | 69.232 | 1.583 | 66.027 | 72.437 |
|  |  | 30 | 72.151 | 2.127 | 67.845 | 76.456 |
|  |  | 40 | 75.273 | 3.179 | 68.837 | 81.710 |
|  |  | 50 | 79.180 | 4.764 | 69.535 | 88.825 |
|  |  | 60 | 85.302 | 7.531 | 70.055 | 100.548 |
| CSA | 5 | 20 | 69.253 | 1.640 | 65.947 | 72.559 |
|  |  | 30 | 71.883 | 1.787 | 68.282 | 75.484 |
|  |  | 40 | 74.784 | 2.557 | 69.631 | 79.937 |
|  |  | 50 | 78.561 | 3.977 | 70.546 | 86.576 |
|  |  | 60 | 85.069 | 6.842 | 71.280 | 98.857 |
| CSA | 6 | 20 | 74.361 | 4.430 | 65.449 | 83.272 |
|  |  | 30 | 79.982 | 7.281 | 65.335 | 94.630 |
|  |  | 40 | 86.097 | 10.999 | 63.970 | 108.225 |
|  |  | 50 | 93.534 | 15.997 | 61.352 | 125.716 |
|  |  | 60 | 103.908 | 23.606 | 56.419 | 151.397 |
| CSA | 7 | 20 | 70.060 | 1.220 | 67.601 | 72.519 |
|  |  | 30 | 73.393 | 1.469 | 70.433 | 76.353 |
|  |  | 40 | 76.795 | 1.992 | 72.780 | 80.809 |
|  |  | 50 | 80.645 | 2.751 | 75.100 | 86.189 |
|  |  | 60 | 85.489 | 3.839 | 77.753 | 93.225 |
| CSA | 8 | 20 | 71.322 | 1.198 | 68.903 | 73.741 |
|  |  | 30 | 72.564 | 1.001 | 70.542 | 74.586 |
|  |  | 40 | 73.676 | 0.938 | 71.781 | 75.570 |
|  |  | 50 | 74.790 | 1.011 | 72.750 | 76.831 |
|  |  | 60 | 76.110 | 1.243 | 73.599 | 78.621 |
| CSA | 9 | 20 | 65.927 | 1.829 | 62.233 | 69.622 |
|  |  | 30 | 70.131 | 2.613 | 64.854 | 75.409 |
|  |  | 40 | 74.421 | 3.971 | 66.401 | 82.441 |
|  |  | 50 | 79.350 | 5.818 | 67.601 | 91.100 |
|  |  | 60 | 85.746 | 8.467 | 68.645 | 102.846 |
| CSA | 10 | 20 | 66.061 | 1.501 | 63.030 | 69.092 |
|  |  | 30 | 68.389 | 1.311 | 65.741 | 71.036 |
|  |  | 40 | 70.630 | 1.600 | 67.400 | 73.861 |
|  |  | 50 | 73.051 | 2.260 | 68.487 | 77.615 |
|  |  | 60 | 75.941 | 3.256 | 69.365 | 82.517 |
| CSA | 11 | 20 | 71.168 | 1.073 | 69.002 | 73.335 |
|  |  | 30 | 73.516 | 1.015 | 71.467 | 75.566 |
|  |  | 40 | 75.837 | 1.266 | 73.281 | 78.394 |
|  |  | 50 | 78.401 | 1.763 | 74.839 | 81.962 |
|  |  | 60 | 81.549 | 2.518 | 76.464 | 86.634 |
| CAL | 1 | 20 | 65.270 | 2.628 | 59.896 | 70.644 |
|  |  | 30 | 67.216 | 3.360 | 60.343 | 74.089 |
|  |  | 40 | 69.219 | 4.255 | 60.516 | 77.922 |
|  |  | 50 | 71.422 | 5.338 | 60.505 | 82.339 |
|  |  | 60 | 74.014 | 6.701 | 60.310 | 87.719 |
| CAL | 2 | 20 | 60.823 | 1.209 | 58.360 | 63.286 |
|  |  | 30 | 62.208 | 0.983 | 60.207 | 64.210 |
|  |  | 40 | 63.498 | 1.148 | 61.160 | 65.837 |
|  |  | 50 | 64.850 | 1.608 | 61.575 | 68.125 |
|  |  | 60 | 66.405 | 2.291 | 61.738 | 71.072 |
| CAL | 3 | 20 | 64.795 | 1.900 | 60.948 | 68.641 |
|  |  | 30 | 67.888 | 1.486 | 64.878 | 70.897 |
|  |  | 40 | 70.499 | 1.387 | 67.691 | 73.307 |
|  |  | 50 | 73.041 | 1.594 | 69.815 | 76.267 |
|  |  | 60 | 75.999 | 2.119 | 71.709 | 80.290 |
| CAL | 4 | 20 | 66.225 | 22.505 | 20.384 | 112.067 |
|  |  | 30 | 70.240 | 28.706 | 11.767 | 128.713 |
|  |  | 40 | 73.789 | 34.447 | 3.622 | 143.957 |
|  |  | 50 | 77.098 | 40.000 | -4.379 | 158.576 |
|  |  | 60 | 80.284 | 45.516 | -12.430 | 172.997 |
| CAL | 5 | 20 | 61.714 | 1.619 | 58.438 | 64.991 |
|  |  | 30 | 63.574 | 1.187 | 61.170 | 65.977 |
|  |  | 40 | 65.212 | 1.140 | 62.904 | 67.520 |
|  |  | 50 | 67.023 | 1.521 | 63.945 | 70.102 |
|  |  | 60 | 69.756 | 2.510 | 64.675 | 74.838 |
| CAL | 6 | 20 | 57.967 | 2.352 | 53.193 | 62.742 |
|  |  | 30 | 61.100 | 1.537 | 57.980 | 64.220 |
|  |  | 40 | 63.124 | 1.203 | 60.682 | 65.567 |
|  |  | 50 | 64.951 | 1.219 | 62.477 | 67.426 |
|  |  | 60 | 66.984 | 1.607 | 63.721 | 70.246 |
| CAL | 7 | 20 | 67.160 | 3.305 | 60.428 | 73.892 |
|  |  | 30 | 69.896 | 4.471 | 60.789 | 79.003 |
|  |  | 40 | 72.523 | 5.701 | 60.910 | 84.135 |
|  |  | 50 | 75.176 | 7.019 | 60.879 | 89.473 |
|  |  | 60 | 77.962 | 8.466 | 60.717 | 95.207 |
| CAL | 8 | 20 | 56.658 | 5.528 | 45.435 | 67.881 |
|  |  | 30 | 62.421 | 3.981 | 54.340 | 70.502 |
|  |  | 40 | 67.223 | 3.723 | 59.664 | 74.782 |
|  |  | 50 | 72.531 | 5.078 | 62.223 | 82.839 |
|  |  | 60 |  |  |  |  |
| CAL | 9 | 20 | 63.481 | 2.449 | 58.493 | 68.469 |
|  |  | 30 | 66.473 | 3.519 | 59.305 | 73.640 |
|  |  | 40 | 69.766 | 5.054 | 59.471 | 80.061 |
|  |  | 50 | 73.989 | 7.283 | 59.153 | 88.825 |
|  |  | 60 | 80.857 | 11.279 | 57.883 | 103.830 |
| CAL | 10 | 20 | 63.714 | 3.239 | 57.139 | 70.290 |
|  |  | 30 | 67.101 | 4.687 | 57.587 | 76.616 |
|  |  | 40 | 70.927 | 6.747 | 57.231 | 84.623 |
|  |  | 50 | 75.797 | 9.699 | 56.107 | 95.488 |
|  |  | 60 | 83.233 | 14.657 | 53.477 | 112.989 |
| CAL | 11 | 20 | 59.714 | 1.991 | 55.684 | 63.745 |
|  |  | 30 | 62.057 | 2.013 | 57.983 | 66.131 |
|  |  | 40 | 64.864 | 2.884 | 59.025 | 70.702 |
|  |  | 50 | 70.211 | 5.569 | 58.937 | 81.484 |
|  |  | 60 |  |  |  |  |
| CAL | 12 | 20 | 65.970 | 1.198 | 63.545 | 68.395 |
|  |  | 30 | 67.352 | 1.866 | 63.575 | 71.129 |
|  |  | 40 | 69.077 | 3.395 | 62.205 | 75.950 |
|  |  | 50 | 71.785 | 6.119 | 59.397 | 84.174 |
|  |  | 60 | 86.783 | 23.390 | 39.432 | 134.134 |
| CAL | 13 | 20 | 64.856 | 1.214 | 62.391 | 67.321 |
|  |  | 30 | 65.395 | 2.643 | 60.029 | 70.762 |
|  |  | 40 | 65.998 | 6.460 | 52.883 | 79.114 |
|  |  | 50 |  |  |  |  |
|  |  | 60 |  |  |  |  |
| CAL | 14 | 20 | 65.050 | 1.688 | 61.622 | 68.477 |
|  |  | 30 | 67.353 | 1.804 | 63.690 | 71.015 |
|  |  | 40 | 70.244 | 3.315 | 63.514 | 76.974 |
|  |  | 50 |  |  |  |  |
|  |  | 60 |  |  |  |  |
| CAL | 15 | 20 | 66.479 | 1.511 | 63.434 | 69.524 |
|  |  | 30 | 68.750 | 1.305 | 66.119 | 71.380 |
|  |  | 40 | 70.731 | 1.214 | 68.284 | 73.178 |
|  |  | 50 | 72.660 | 1.234 | 70.173 | 75.147 |
|  |  | 60 | 74.806 | 1.385 | 72.014 | 77.598 |
| CAL | 16 | 20 | 65.600 | 0.738 | 64.090 | 67.110 |
|  |  | 30 | 66.358 | 1.014 | 64.284 | 68.431 |
|  |  | 40 | 67.237 | 1.453 | 64.264 | 70.209 |
|  |  | 50 | 68.394 | 2.106 | 64.088 | 72.701 |
|  |  | 60 | 70.351 | 3.285 | 63.633 | 77.069 |
| CAL | 17 | 20 | 68.265 | 2.800 | 62.580 | 73.950 |
|  |  | 30 | 71.030 | 3.746 | 63.425 | 78.635 |
|  |  | 40 | 74.104 | 4.987 | 63.980 | 84.228 |
|  |  | 50 | 77.870 | 6.666 | 64.337 | 91.402 |
|  |  | 60 | 83.162 | 9.222 | 64.440 | 101.884 |
| CAL | 18 | 20 | 66.086 | 5.105 | 55.722 | 76.450 |
|  |  | 30 | 66.726 | 8.070 | 50.344 | 83.108 |
|  |  | 40 |  |  |  |  |
|  |  | 50 |  |  |  |  |
|  |  | 60 |  |  |  |  |
| CAL | 19 | 20 | 65.023 | 0.872 | 63.247 | 66.798 |
|  |  | 30 | 65.954 | 0.827 | 64.269 | 67.639 |
|  |  | 40 | 66.705 | 1.018 | 64.631 | 68.778 |
|  |  | 50 | 67.427 | 1.317 | 64.743 | 70.111 |
|  |  | 60 | 68.316 | 1.760 | 64.730 | 71.902 |
| CAL | 20 | 20 | 62.275 | 1.739 | 58.744 | 65.806 |
|  |  | 30 | 65.203 | 1.879 | 61.389 | 69.017 |
|  |  | 40 | 68.084 | 2.451 | 63.109 | 73.059 |
|  |  | 50 | 71.322 | 3.381 | 64.458 | 78.187 |
|  |  | 60 | 75.459 | 4.791 | 65.733 | 85.186 |
